# Electrical pulse stimulation reflecting the episodic nature of real‐life exercise modulates metabolic and secretory profile of primary human myotubes

**DOI:** 10.1002/2211-5463.70114

**Published:** 2025-09-09

**Authors:** Klára Gabrišová, Tímea Kurdiová, Daria Barkova, Natália Pálešová, Jana Babulicová, Silvia Tyčiaková, Marta Novotová, Mária Balážová, Miroslav Sabo, Václav Pustka, Jozef Ukropec, Barbara Ukropcová

**Affiliations:** ^1^ Department of Metabolic Disease Research, Institute of Experimental Endocrinology, Biomedical Research Center Slovak Academy of Sciences Bratislava Slovakia; ^2^ Department of Molecular Oncology, Cancer Research Institute, Biomedical Research Center Slovak Academy of Sciences Bratislava Slovakia; ^3^ Department of Cellular Cardiology, Institute of Experimental Endocrinology, Biomedical Research Center Slovak Academy of Sciences Bratislava Slovakia; ^4^ Department of Membrane Biochemistry, Institute of Animal Biochemistry and Genetics, Centre of Biosciences Slovak Academy of Sciences Bratislava Slovakia; ^5^ Department of Bioinformatics, Institute of Experimental Endocrinology, Biomedical Research Center Slovak Academy of Sciences Bratislava Slovakia; ^6^ Central European Institute of Technology, Masaryk University Brno Czech Republic; ^7^ Institute of Pathophysiology, Faculty of Medicine Comenius University Bratislava Slovakia

**Keywords:** electrical pulse stimulation, extracellular vesicles, glucose and fatty acid metabolism, growth differentiation factor 11, human primary muscle cell culture, myokines

## Abstract

Electrical pulse stimulation (EPS) represents a useful tool to study exercise‐related adaptations of muscle cells *in vitro*. Here, we examine the metabolic and secretory response of primary human muscle cells from metabolically healthy individuals to the EPS protocol reflecting the episodic nature of real‐life exercise training. This intermittent EPS protocol alternates high‐frequency stimulation periods with low‐frequency resting periods. Continuous EPS was used as a comparator. Radiometric assessment of glucose and fatty acid metabolism was complemented by examination of mitochondrial OxPHOS proteins, fiber‐type markers, and the release of selected myokines and extracellular vesicles into the media. Both EPS protocols facilitated glycogen synthesis and incomplete fatty acid oxidation (intermediary metabolites accumulation), while complete glucose and fatty acid oxidation (CO_2_ production) was increased only after the intermittent stimulation. Continuous stimulation elicited robust release of the contraction‐regulated myokines (IL6, IL8) into the media. Both EPS protocols increased expression of oxidative fiber‐type markers (*MYH2, MYH7*), while inducing protein expression of a putative myokine, growth differentiation factor11 (GDF11) and a release of extracellular vesicles into the media. In conclusion, intermittent electrical pulse stimulation enhanced the rate of complete glucose and fatty acid oxidation in differentiated muscle cells from metabolically healthy individuals, while it was comparable to continuous stimulation in modulating markers of oxidative fibers and a putative myokine GDF11, and less effective in stimulating the release of myokines IL6, IL8, and extracellular vesicles into the media. Intermittent EPS—a protocol mimicking the episodic nature of exercise—can be used for studying metabolism and the secretome of skeletal muscle cells *in vitro*.

## Graphical abstract

Here, we introduced an intermittent electrical stimulation protocol mimicking the episodic nature of real‐life exercise *in vitro* by alternating low‐ and high‐frequency stimulation. In comparison with widely used continuous stimulation, it enhanced the rate of glucose and fatty acid oxidation, but not the myokine release. Both protocols increased the rate of glycogen synthesis, oxidative fiber‐type markers, and extracellular vesicles' release.

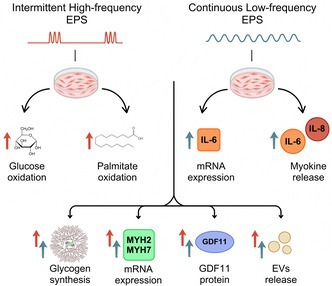

**Abbreviations**
ASMs: acid‐soluble metabolites
CON: control
CS: continuous electrical pulse stimulation
DAG: diacylglycerol
EPS: electrical pulse stimulation
EVs: extracellular vesicles
FOx: fatty acid oxidation
GDF11: growth differentiation factor 11
IL6: interleukin 6
IL8: interleukin 8
Ins: insulin
IS: intermittent electrical pulse stimulation
NTA: nanoparticle tracking analysis
OxPHOS: oxidative phosphorylation
PGC1α: peroxisome proliferator‐activated receptor gamma coactivator 1‐alpha
PL: phospholipid
TAG: triacylglycerol
TEM: transmission electron microscopy

Exercise is widely recognized for its beneficial effects on human health, providing cardiopulmonary fitness, metabolic flexibility, and better quality of life [1, 2, 3]. However, the intricate molecular mechanisms underlying the muscle‐specific and systemic changes related to exercise (repeated muscle contractions) remain incompletely understood.

Strategies used to mimic effects of exercise in cultured muscle cells involve treatments with bioactive molecules targeting exercise‐activated signaling pathways, including AMP‐activated protein kinase (AMPK) agonist AICAR, protein kinase A activator forskolin, and/or treatment with caffeine or ionomycin aimed at elevating levels of intracellular Ca^+^ [4, 5, 6]. However, these approaches target specific molecular pathways and may not fully recapitulate the complexity of the response to exercise (repeated muscle contraction).

In contrast, electrical pulse stimulation (EPS) offers a more physiologically relevant approach to study the complex effects of exercise *in vitro*. Application of controlled electrical pulses leads to depolarization of the muscle cell membrane, triggering excitation–contraction coupling resulting in repeated contractions of cultured differentiated skeletal muscle cells, myotubes [7]. This process activates multiple downstream molecular pathways involved in immediate and long‐term adaptive response to exercise. The physiological relevance of this method has been demonstrated through the recapitulation of key exercise‐induced responses, such as sarcomere assembly [8], induction of cytosolic Ca^2+^‐transients [9], activation of AMPK [8, 10, 11], increased glucose uptake [7, 8, 11], enhanced glucose [8] and fatty acid oxidation [7, 8, 12], induction of mitochondrial biogenesis markers (PGC1α) [8, 13], production and release of myokines such as interleukin 6 (IL6) or interleukin 8 (IL8) [7, 8] or extracellular vesicles (EVs) [14, 15], an integral part of muscle secretome, which contains a spectrum of bioactive molecules involved in the exercise‐induced interorgan crosstalk [16].

Previous research highlighted EPS as a useful tool to study contraction‐related changes in secretome and transcriptome of muscle cells [13, 17], to examine exercise‐induced cell‐to‐cell communication [10], and to investigate effects of exercise on muscle and whole‐body energy metabolism [7, 8, 18]. Growth Differentiation Factor 11 (GDF11) is a putative myokine [19] with the capacity to stimulate proliferative potential of stem cells in skeletal muscle, heart, and brain [20]. Previously, we described exercise‐induced downregulation of GDF11 in cerebrospinal fluid of healthy young volunteers, [21] and here, we examined the hypothesis that EPS modulates production and release of GDF11 in muscle cells, which would support the evidence that GDF11 is an exercise‐regulated myokine. Furthermore, it is plausible to think that the adaptive response to EPS can be linked to the metabolic phenotypes of muscle cells' donors [18, 22, 23] and their response to the training intervention *in vivo* [24].

Numerous studies utilized several EPS protocols, aiming to replicate the molecular signatures associated with different exercise modalities through modulation of EPS parameters, such as voltage, frequency, pulse duration, and total stimulation time [25, 26]. Among the existing approaches, continuous chronic low‐frequency (≤ 1 Hz) EPS of human myotubes lasting for 24–48 h, aiming to mimic chronic exhaustive exercise, is commonly used [7, 12, 13, 18, 27, 28]. However, many adaptations occur when repeated short‐term exercise with higher intensity is combined with periods of inactivity, enabling muscle recovery. Therefore, treatments that mimic continuous exhaustive exercise—rather than reflecting the episodic nature of real‐life training—may be less effective in inducing the appropriate metabolic and secretory adaptations. Here, we designed a stimulation protocol, where lower and higher intensity (frequency) of electrical stimulation alternate, to mimic an exercise regime where repeated high‐intensity (frequency) exercise intervals are combined with lower intensity “recovery” periods (intermittent EPS).

The primary goal of this study was to examine the differences in energy metabolism and secretory function elicited by intermittent and continuous EPS protocols in differentiated muscle cells from metabolically healthy men, with the intention to create a more physiological model of exercise *in vitro*. Therefore, we examined the EPS—induced changes in glucose and fatty acid oxidation rate, the rate of glycogen and lipid synthesis, mitochondrial OxPHOS proteins, muscle fiber‐type markers, and the secretion of well‐established contraction‐regulated myokines such as IL6 and IL8. Moreover, we examined the EPS‐related release of extracellular vesicles and expression of the GDF11.

## Materials and methods

### Ethics approval

The protocol of the study, which included the collection of skeletal muscle biopsies and muscle cell cultures used in this report, was approved by the Ethics Committee of the University Hospital Bratislava, Comenius University Bratislava on 01.07.2008 (protocol No 2.1.1‐6). The study conforms to the standards of the 2000 Helsinki declaration, including its recent amendments. All volunteers signed a written informed consent prior to entering the study. Consent included the use of cultured muscle cells for further research.

### Human primary skeletal muscle cell culture

Samples of skeletal muscle (*m. vastus lateralis*) were taken from men without obesity and with normal glucose metabolism, insulin sensitivity, and plasma lipids (Table 1). Biopsies were performed by the Bergström technique after an overnight fast under local anesthesia and cultured as previously described in [5]. Briefly, satellite cells (quiescent mononuclear muscle cells) were isolated by trypsin digestion of the freshly obtained and finely minced muscle tissue, preplated on an uncoated Petri dish, and incubated for 60 min (37 °C, 5% CO_2_) in Dulbecco's modified Eagle's medium (DMEM low glucose: 5.55 mm; Lonza, Basel, Switzerland) with 15% fetal bovine serum (FBS, Lonza) to remove attached fibroblasts. Tissue cuts were transferred to a T‐25/collagen‐coated flask and incubated in DMEM with 15% FBS, L‐glutamine (Lonza), human epidermal growth factor (Sigma‐Aldrich, Burlington, MA, USA), dexamethasone (Sigma‐Aldrich), BSA (Sigma‐Aldrich), fetuin (Sigma‐Aldrich), gentamycin (Lonza) and fungizone (Lonza). Cells were passaged once, frozen (−1 °C·min^−1^, Mr. Frosty; Thermo Fisher Scientific, Waltham, MA, USA) and stored in liquid nitrogen. When ready for an experiment, cells were thawed and seeded into non‐coated 6‐well (for RNA, proteins) and 12‐well (for glucose and fatty acid oxidation, glycogen and lipid synthesis assays) plates (Falcon Becton Dickinson, Franklin Lakes, NJ, USA) at a density of ∼4000 cells per cm^2^. Differentiation was initiated at 80–90% confluence by switching to *α*‐minimum essential medium (α‐MEM, Gibco, Waltham, MA, USA) with 2% FBS (Gibco), 25 pm insulin (Sigma‐Aldrich), fetuin (Gibco), and penicillin–streptomycin (Lonza). Cells were kept in a humidified atmosphere of 5% CO_2_ at 37 °C. In total, we performed four independent experiments. In each experiment, we used cells from one specific donor. Glucose and fatty acid metabolism were examined radiometrically in triplicate wells (Table S1). In the radiometric analyses, we present all the datapoints corresponding to each specific well on the culture plate.

**Table 1:** Clinical characteristics of the cells' donors. Body composition was assessed by bioelectric impedance. 2nd degree relative; BMI, body mass index; HDL, high‐density lipoprotein; insulin sensitivity index was measured by euglycemic hyperinsulinemic clamp (*M*‐value normalized to insulinemia); HOMA‐IR, homeostatic model assessment of insulin resistance; oGTT, 2 h oral glucose tolerance test; T2DM, type 2 diabetes mellitus.

	Donor 1	Donor 2	Donor 3	Donor 4
Gender	Man	Man	Man	Man
Age [years]	28	34	30	56
BMI [kg/m^2^]	24.2	21.8	25.2	23.5
Body weight [kg]	80.0	80.5	89.0	72.4
Waist circumference [cm]	87	84	105	72
Body fat [%]	19.6	10.1	19.7	15.2
Lean body mass [kg]	64.3	72.4	71.5	61.4
Fasting glucose [mmol/L]	5.01	4.34	4.80	4.72
2 h glucose oGTT [mmol/L]	5.80	4.60	3.80	4.06
HOMA‐IR	0.45	0.39	0.43	0.42
Triacylglycerols [mmol/L]	0.61	0.82	0.90	0.84
Total cholesterol [mmol/L]	4.02	3.95	3.75	4.20
HDL‐cholesterol [mmol/L]	1.72	1.41	1.11	1.25
Insulin sensitivity index [mg.kg^−1^.min^−1^/μU.mL^−1^]	0.21	0.18	0.20	0.20
Family history–T2DM	2nd degree	No	2nd degree	No
Family history–obesity	No	2nd degree	2nd degree	No

### Electrical pulse stimulation

**Fig. 1:**
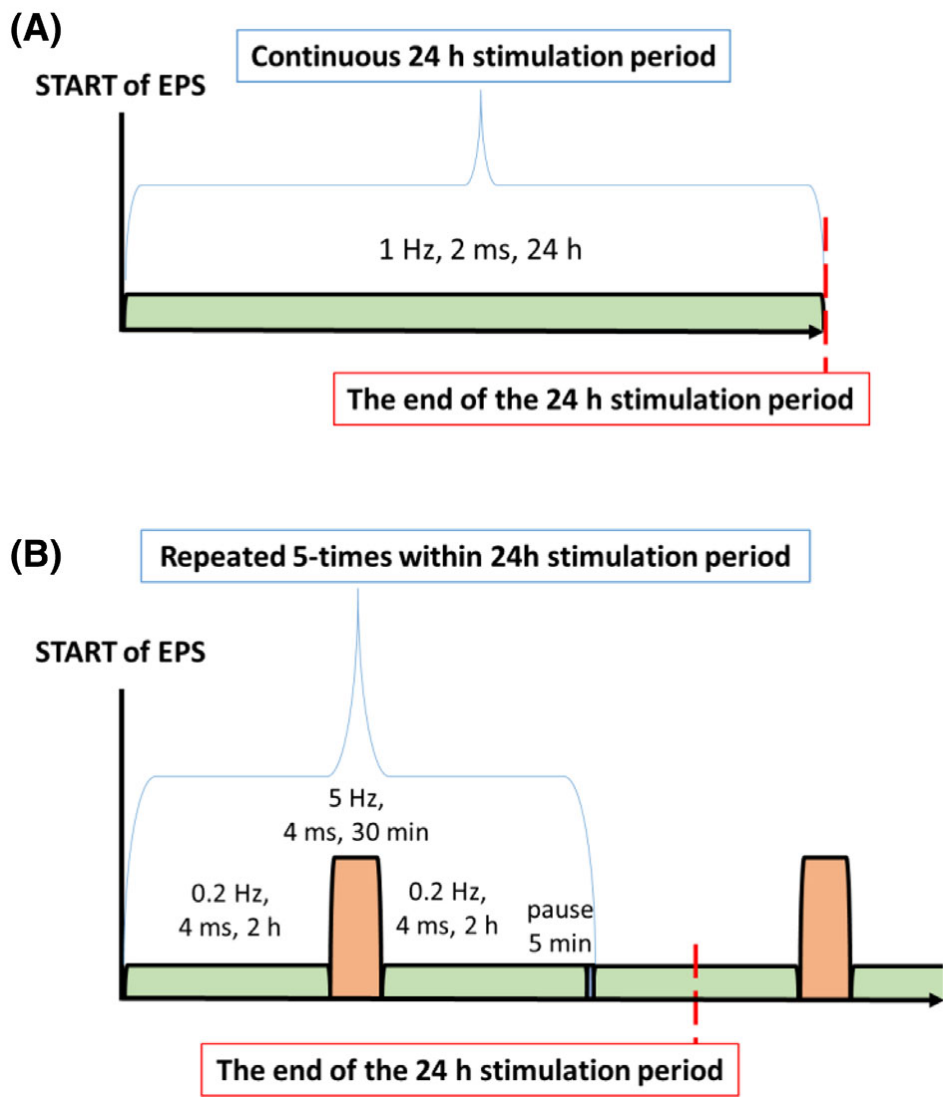
Schematic representation of the protocols for continuous (A) and intermittent (B) electrical pulse stimulation (EPS).

To induce repeated and well‐controlled contractions of myotubes in culture, electrical pulse stimulation was performed using the C‐Pace EP cell culture stimulator with C‐dish and carbon electrodes (IonOptix, Westwood, MA, USA). After 5 days of cell differentiation, new differentiation media were added to plates used to examine glucose and fatty acid metabolism, and gene/protein expression, while fasting media (α‐MEM) without FBS and fetuin were used to study EPS‐induced production of myokines (IL6, IL8, and GDF11) and EVs. In brief, fully differentiated myotubes were electrically stimulated, implementing two distinct EPS protocols: (i) continuous 24 h stimulation aimed at mimicking constant low‐intensity exhausting exercise (frequency 1 Hz, pulse duration 2 ms), and (ii) intermittent 24 h stimulation where 30‐min intervals of higher frequency stimulation (5 Hz, pulse duration 4 ms) were alternated with 4 h of low subthreshold frequency stimulation (0.2 Hz), allowing muscle cell recovery, while using the same electric field gradient of 1.1 V per cm^2^ (11.5 V for six‐well plates and 4.0 V for 12‐well plates). Each stimulation cycle of the intermittent stimulation protocol (0.2 Hz–5 Hz–0.2 Hz) was concluded by a 5‐min resting period. The cycle was repeated five times in a 24 h period, and cells were harvested immediately after completing the 24 h EPS protocol, within the period of 0.2 Hz stimulation, precisely 3 h after the last high‐frequency (5 Hz) stimulation period (Fig. 1).

Control myotubes were treated similar to EPS‐stimulated myotubes, except that the electrodes immersed in the media were not connected to the pulse generator [25]. Cells and media were harvested for protein, mRNA, and EVs isolation and analyses immediately after concluding the 24 h EPS protocol, that is, at 7th day of differentiation. An identical timeline was applied for the radiometric assays examining glucose and fatty acid oxidation, glycogen, and lipid synthesis.

### Effects of electric pulse stimulation on glucose oxidation and glycogen synthesis

Following the electric pulse stimulation, the cells were first preincubated (90 min) in glucose‐free, serum‐free medium. This was followed by an acute glucose challenge, that is, 3 h incubation with D[U‐^14^C] glucose (0.2 mCi·mL^−1^; PerkinElmer®, Waltham, MA, USA) diluted in 1 mm of nonlabeled (cold) glucose (Sigma‐Aldrich). The effect of insulin on glucose metabolism was examined (50 nm insulin Humulin; Eli Lilly, Indianapolis, IN, USA). Next, the conditioned media were transferred into the 48‐well custom‐made CO_2_‐trapping plate made of the chemically inert PTFE. The amount of ^14^CO_2_ released from the media by 70% perchloric acid (Merck, Rahway, NJ, USA) was trapped in 1 M NaOH (Merck). To measure the rate of glycogen synthesis, cells were washed twice and harvested in 1 M KOH (Sigma‐Aldrich). Cold glycogen (2.1 mg; Sigma‐Aldrich) was added to 200 μL of cell lysates and heated at 70 °C for 20 min. Following incubation, 500 μL of ice‐cold 100% ethanol (Centralchem, Bratislava, Slovakia) was added to precipitate glycogen. The tubes were centrifuged (20 min/8280 ***g***), the pellet was washed with 70% ethanol. The glycogen precipitate was resuspended in distilled water. The supernatant (rest of cell lysate) was used to measure protein content for data normalization (BCA; Thermo‐Fisher Scientific). The amount of the accumulated ^14^CO_2_ and ^14^C‐glycogen was measured in EcoLite(+)™ Liquid Scintillation Cocktail (MP Biomedicals, Santa Ana, CA, USA) by using a liquid scintillation analyzer (TriCarb2910TR; Perkin‐Elmer). Rates of glucose oxidation and glycogen synthesis were adjusted for specific activity to account for dilution of [^14^C] glucose with nonlabeled (cold) glucose and glycogen. The total glucose disposal rate was calculated as a sum of ^14^C‐glucose oxidation rate (rate of CO_2_ production) and glycogen incorporation rate normalized to protein content in each specific well.

### Effects of electric pulse stimulation on fatty acid oxidation and lipid synthesis

Immediately after concluding the electric pulse stimulation protocol, the cells were preincubated (90 min) with glucose‐free and serum‐free media. For the subsequent 3 h, cells were exposed to saturated fatty acid, [1‐^14^C]‐palmitate (0.1 mCi·mL^−1^; American Radiolabeled Chemicals Inc., St. Louis, MO, USA), coupled to fatty acid‐free BSA (Sigma‐Aldrich). Following incubation, media were transferred into the 48‐well custom‐made CO_2_‐trapping plate made of the chemically inert PTFE. The amount of ^14^CO_2_ released from media by perchloric acid was collected in 1 m NaOH. Acidified media were spun twice at (15 000 RPM, 10 min at 4 °C) and activity of the supernatant containing intermediate FOx metabolites (acid‐soluble metabolites, ASMs) was measured using liquid scintillation cocktail (EcoLite+^TM^; MP Biomedicals) and TriCarb2910TR scintillation counter (Perkin‐Elmer). Cells were washed twice (ice‐cold phosphate‐buffered saline), harvested (0.25 mL, 0.05% SDS), and used to measure protein content for data normalization (BCA; Thermo‐Fisher Scientific). All the assays were performed in triplicates. Total fatty acid oxidation (Total FOx) rate was calculated as a sum of ^14^C‐CO_2_ and ^14^C‐ASMs.

Incorporation of labeled palmitate into major lipid species (phospholipids, diacylglycerols, and triacylglycerols) was detected by thin layer chromatography (TLC). Neutral lipids were extracted from myotubes exposed for 3 h to [^14^C]‐palmitate (acute palmitate challenge), using the protocol described in [29]. Dried lipid fractions were dissolved in chloroform:methanol (2 : 1; v : v), and aliquots were applied to TLC–silica gel 60 plates (Merck). Neutral lipids were separated by the ascending two‐step TLC. Individual spots representing phospholipids, diacylglycerols, and triacylglycerols were identified by lipid standards run on the same plate and scraped off into the scintillation vials to measure the activity of incorporated [^14^C]‐palmitate (as specified above). Data were normalized to the protein content of the corresponding wells.

### RNA isolation and qPCR

Total RNA was isolated from myotubes (Qiazol; Qiagen, Germantown, MD, USA) and treated with DNase‐I (New England Biolabs, Ipswich, MA, USA). Quantity and purity of RNA were determined spectrophotometrically (NanoDrop‐2000c; Thermo‐Fisher Scientific). RNA was reverse‐transcribed to cDNA (High‐Capacity cDNA Reverse Transcritption Kit; Thermo‐Fisher Scientific) and used for qPCR (Quant‐Studio‐5; Thermo‐Fisher Scientific) with PowerUp™ SYBR™ Green Master Mix (Thermo‐Fisher Scientific) and specific primer sets (Table S2). *Ribosomal β2‐microglobulin* and *GAPDH* were used as internal references. Relative gene expression was calculated (Δ*C*_T_ method).

### Protein extraction and immunoblotting

Cells were harvested in lysis buffer (50 mm Tris, 150 mm NaCl, 1% Triton X100; pH 8.0) supplemented with Complete Roche (Roche Diagnostics GmbH, Rotkreuz, Switzerland), Phosphatase Inhibitor 2 and 3 (Sigma‐Aldrich). After sonication (3 × 5 min) in ultrasound water bath (5–10 °C), cell lysates were centrifuged (10 000 ***g***, 15 min, 4 °C) and the supernatant was used for immunoblotting and to determine protein concentration (Pierce™ BCA kit; Thermo‐Fisher Scientific).

Thirty μg of proteins was mixed with an appropriate amount of 4× SDS sample loading buffer, incubated for 10 min at 37 °C (OxPHOS complexes, GDF11, heat shock protein 90–HSP90). Proteins were separated in a 10%‐SDS/polyacrylamide gel and blotted into a PVDF membrane (Millipore, Darmstadt, Germany). After blocking (Odyssey blocking buffer; LI‐COR, Lincoln, NB, USA) membranes were incubated overnight with an OxPHOS human primary antibody cocktail, recognizing the Complex I subunit (NDUFB8), Complex II subunit (SDHB), Complex III subunit (UQCRC2), Complex IV subunit (MTCO1) and Complex V alpha subunit (ATP synthase) (Ab110413, 1 : 250; Abcam, Cambridge, UK), GDF11 antibody (Ab71347, 1 : 1000; Abcam), and HSP90 antibody (Ab53497, 1 : 5000; Abcam). Appropriate secondary antibodies (IRDye 680RD and/or 800CW, LI‐COR, 1 : 10000) were used to visualize protein content with the Odyssey IR imaging system (LI‐COR). Protein ladder 10–180 kDa (Thermo‐Fisher‐Scientific) was used. HSP90 was used as a loading control.

### Measurement of myokines (IL6, IL8) and activity of lactate dehydrogenase (LDH) in EPS‐conditioned media

EPS‐conditioned media (FBS‐free and fetuin‐free) was centrifuged (500 ***g***, 10 min, 4 °C) to remove contaminating cells, and the cell‐free supernatant was recentrifuged at a higher speed (1500 ***g***, 10 min, 4 °C). EPS‐induced release of myokines (IL6 and IL8) into the media was determined by Human IL6 and IL8 Standard ELISA development kits (PeproTech) according to the manufacturer's instructions. Data were normalized to the total protein content measured in the cell lysates of the corresponding wells with Pierce™ BCA kit (Thermo‐Fisher Scientific). The activity of LDH released during EPS into conditioned media, which served as an indicator of potential EPS‐induced myotube damage, was determined colorimetrically by the Alinity c Lactate Dehydrogenase Kit (Abbott, Lake Forest, IL, USA) and normalized to the total protein content of the corresponding wells determined by Pierce™ BCA kit (Thermo‐Fisher Scientific).

### Isolation of extracellular vesicles from EPS‐conditioned media

The 50 mL of cell culture medium collected after EPS stimulation was immediately centrifuged at 4 °C, sequentially 3 × 10 min, at increasing speeds (300 ***g***, 1500 ***g***, and 2500 ***g***) to remove contaminating cells and large cell debris. After each step, the supernatant was carefully transferred to a new tube. Cell (debris) free media were concentrated using Amicon Ultra‐15 filters (UFC810096; Millipore). ExoQuick‐TC^®^ ULTRA for Tissue Culture Media (Systems Biosciences, Palo Alto, CA, USA) was used to isolate EVs according to the manufacturer's instructions. The EVs containing pellet (precipitated EVs) was resuspended in nanoparticle free PBS (Gibco) and stored at −80 °C in several 50 μL aliquots until further analyses.

### EVs characterization by nanoparticle tracking analysis and transmission electron microscopy

Concentration and size of EVs were assessed using nanoparticle tracking analysis (NTA, NanoSight NS500, Malvern Panalytical Ltd., Malvern, UK). This instrument allows for the examination of Brownian movement of nanoparticles and calculation of their concentration and size distribution [30]. Polystyrene beads (100 nm, Malvern Panalytical Ltd.) were used for the size calibration. For each measurement, 600 μL of the 60× diluted sample was used, and the experiment was performed in triplicates, that is, three 60 s videos of Brownian motion were analyzed. Serial dilutions were tested for EPS‐conditioned media with the aim of reaching the working concentration range of 10^7^ to 10^9^ particles per mL. Samples were measured at a temperature of 24 °C. Analysis was performed with the in‐built NanoSight Software NTA 2.3. Concentration of nanoparticles was normalized to the total protein content of the cells (Pierce™ BCA kit; Thermo‐Fisher Scientific).

Isolated EVs (5 μL) were mixed with 5 μL of 4% paraformaldehyde and incubated at 4 °C for 30 min. A 5–7 μL of the mixture was applied to a Formvar‐coated copper TEM grid (100 mesh; Sigma‐Aldrich) and dried for 10 min at RT. After removing the excess liquid with filter paper, the samples were contrasted with a 1% aqueous solution of uranyl acetate (5 min) and washed with distilled water. After drying (10 min), the samples were examined with a JEM 1200 electron microscope (Jeol Ltd., Osaka, Japan) at 80 kV. The images were recorded with a CCD camera (Gatan Dual Vision 300 W).

### Proteomic analysis of EVs from EPS‐conditioned media

EV‐contained proteins were extracted from a 50 μL aliquot in an 8 M urea buffer in a thermomixer (Eppendorf ThermoMixer C; Eppendorf, Hamburg, Germany, 15 min, 37 °C, 750 RPM). After that, all samples were centrifuged (15 min, 20 000 ***g***), and the supernatants (approx. 10 μg of total protein) were used for filter‐aided sample preparation (FASP) using 8 m urea‐based buffers for protein washing. Subsequent enzymatic digestion was done with trypsin (sequencing grade; Promega, Madison, WI, USA). The resulting peptides were extracted into LC–MS vials by 2.5% formic acid (FA) in 50% acetonitrile (ACN) and 100% ACN with the addition of polyethylene glycol (final concentration 0.001%) [31] and concentrated in a SpeedVac concentrator (Thermo‐Fisher Scientific).

LC–MS/MS analyses of all peptides were done using the UltiMate 3000 RSLCnano system (Thermo‐Fisher Scientific) connected to the timsTOF Pro mass spectrometer (Bruker, Bremen, Germany). Before LC separation, tryptic digests were online concentrated and desalted using a trapping column (Acclaim PepMap 100 C18, dimensions 300 μm ID, 5 mm long, 5 μm particles; Thermo‐Fisher Scientific). The trap column was then washed with 0.1% TFA and the peptides were eluted in backflush mode from the trapping column onto an analytical column (Aurora C18, 75 μm ID, 250 mm long, 1.7 μm particles; Ion Opticks, Collingwood, VIC, Australia) by a 90‐min gradient program (flow rate 150 nL.min^−1^, 3–42% of mobile phase B; mobile phase A: 0.1% FA in water; mobile phase B: 0.1% FA in 80% ACN; Thermo‐Fisher Scientific) followed by a system wash using 80% of mobile phase B. Equilibration of the trapping column and the analytical column was done before sample injection to the sample loop. The analytical column was installed in the Captive Spray ion source (Bruker; temperatures set to 50 °C) according to the manufacturer's instructions. Spray voltage and sheath gas were set to 1.5 kV and 1, respectively.

MS data were acquired in data‐independent acquisition (DIA) mode with base method m/z range of 100–1700 and 1/k0 range of0.6 to 1.4 V × s × cm^−2^. Enclosed DIAparameters.txt file defined m/z 400–1000 precursor range with equal window size of 21 Th using two steps for each PASEF scan and cycle time of 100 ms locked to 100% duty cycle.

DIA data were processed in DIA‐NN (version 1.8.1) [32] in library‐free mode against the iRT peptide sequences database (Biognosys, Schlieren, Switzerland) and UniProtKB protein database for *Homo sapiens* (https://www.uniprot.org/proteomes/UP000005640); version 2024/03, number of protein sequences: 20598. No optional, but carbamidomethylation as a fixed modification and trypsin/P enzyme with 1 allowed missed cleavage and peptide length 7–30 were set during the library preparation. False discovery rate (FDR) control was set to 1% FDR. MS1 and MS2 accuracies as well as scan window parameters were set based on the initial test searches (median value from all samples ascertained parameter values). MBR was switched on.

Protein MaxLFQ intensities reported in the DIA‐NN main report file were further processed using the software container environment (https://github.com/OmicsWorkflows), version 4.7.7a. The processing workflow is available upon request. Briefly, it covered: (a) removal of low‐quality precursors and contaminant protein groups, (b) precursor intensities normalization by the loessF algorithm, (c) protein group MaxLFQ intensities calculation, and log_2_ transformation. The log_2_‐transformed intensities were categorized into low, middle, and high‐value groups, representing the 0.10, 0.5 (median), and 0.90 percentiles of all nonzero intensities, respectively.

### Statistical analysis

Statistical analyses were performed using GraphPad Prism version 9.5.1 (GraphPad Software, Inc., USA) or RStudio [33, 34]. Outlier detection was performed using the boxplot method, and normality was assessed using the Shapiro–Wilk test. For comparisons among the three conditions (CON, CS, IS), a one‐way ANOVA followed by Tukey's multiple comparisons test was used. For variables that did not meet the assumption of normality, the nonparametric Friedman test was used as an alternative to the parametric ANOVA. Presented data are normalized to the control condition (with electrodes, without EPS) and presented as mean ± SEM. *P* < 0.05 indicates statistical significance and *P* < 0.1 trend. Sample size and specification of the statistical tests are described in the figure legends.

## Results

Both continuous low‐frequency and intermittent high‐frequency electrical pulse stimulation (EPS) protocols elicited visible contractions of myotubes in culture (Videos S1 and S2). The potential cytotoxic effect of the two EPS protocols, indicative of cell lysis or membrane damage, was assessed by measuring LDH activity in the conditioned media. Results showed that neither continuous (CS) nor intermittent stimulation (IS) increased LDH activity as compared to controls (CON) (CS: 2.06 ± 1.36; IS: 2.08 ± 0.72; CON: 2.18 ± 0.67 [μkat/l/ug cell proteins]; *n* = 4; *P* > 0.05), suggesting that neither exercise‐mimicking protocol induces any damage to the differentiated human primary muscle cells in culture.

### Effects of electrical pulse stimulation on metabolism of glucose and fatty acids

**Fig. 2:**
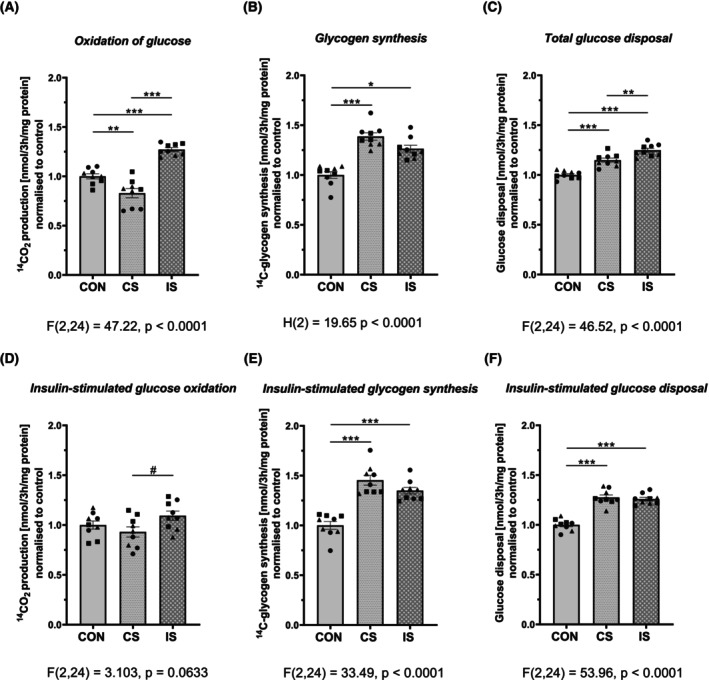
Effects of electrical pulse stimulation (EPS) on glucose metabolism in primary human myotubes. Human myotubes were exposed to continuous (CS) or intermittent (IS) electrical pulse stimulation. The effect of EPS on (A) glucose oxidation, (B) glucose incorporation into glycogen, and (C) the total glucose disposal (oxidative + nonoxidative: CO_2_ + glycogen) was measured. (D–F) depict the effects of CS and IS EPS on the identical parameters of glucose metabolism under insulin‐stimulated conditions. Data are normalized to controls and presented as mean ± SEM of *n* = 9 (3 independent experiments, each with triplicate measurements). Individual cell donors are depicted by different symbols ▲, ●, ■. The difference between control and EPS‐treated myotubes was assessed by one‐way ANOVA with Tukey's multiple comparison test. In the case of non‐normal data distribution (glycogen synthesis) Kruskal‐Wallis test with Dunn's multiple comparison was used. Level of significance: ***P < 0.001, **P < 0.01, *P < 0.05. CON, control; CS, continuous; IS, intermittent EPS.

Intermittent stimulation (IS) increased both the rate of glucose oxidation (CO_2_ production) and glycogen synthesis in differentiated primary muscle cells from metabolically healthy individuals as compared to nonstimulated (CON) cells (Fig. 2A,B). Continuous stimulation (CS), however, increased the rate of glycogen synthesis, but it had no stimulatory effect on the rate of glucose oxidation. Total glucose disposal was therefore increased in response to both EPS protocols (Fig. 2C). Almost identical effects of IS and CS were observed in the insulin stimulated state (Fig. 2D–F). Insulin induced a robust (1.4–1.5 fold) increase in the rate of glycogen synthesis in all experimental conditions.

**Fig. 3:**
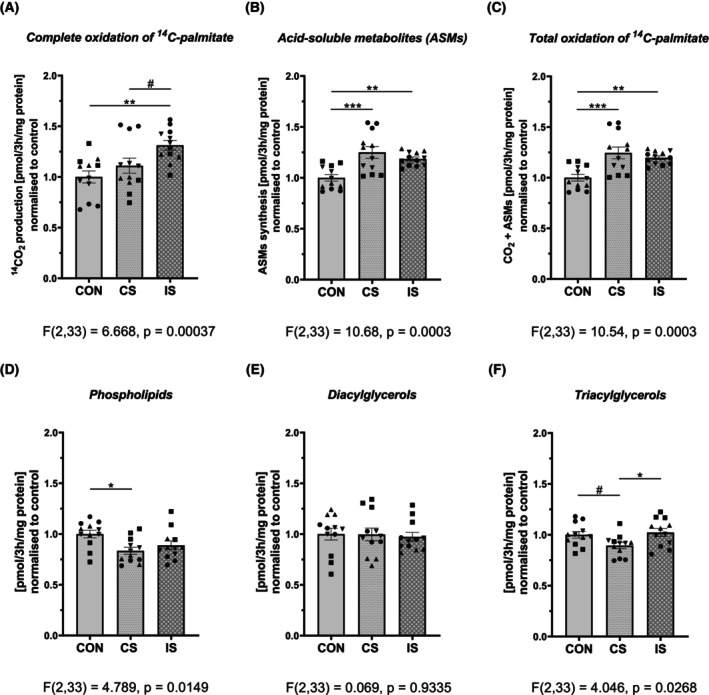
Effects of electrical pulse stimulation (EPS) on lipid metabolism in primary human myotubes. Differentiated human muscle cells were exposed to continuous (CS) or intermittent (IS) EPS. Here we present the effects of EPS on (A) complete fatty acid oxidation (FOx, CO_2_ production), (B) incomplete FOx: the accumulation of the intermediate metabolites of FOx (Acid‐Soluble Metabolites, ASMs), (C) total FOx (FOx = CO_2_ + ASMs) and on the rate of de novo lipid synthesis of (D) phospholipids, (E) diacylglycerols, and (F) triacylglycerols. Data are normalized to controls and are presented as mean ± SEM of *n* = 12 (*n* = 4 independent experiments, each with triplicate measurements). Individual donors were depicted by different symbols ▲, ●, ■, ▼. The difference between control and EPS‐treated myotubes was assessed by one‐way ANOVA with Tukey's multiple comparison test. Level of significance: ***P < 0.001, **P < 0.01, *P < 0.05, and *P* < 0.1 indicates trend. ASMs, acid‐soluble metabolites; CON, control; CS, continuous EPS; FOx, fatty acid oxidation; IS, intermittent EPS.

Intermittent stimulation (IS) of human primary myotubes enhanced complete FOx (CO_2_ production), while in parallel increasing the accumulation of fatty acid intermediate metabolites (ASMs, products of incomplete FOx) and total fatty acid oxidation rate (Fig. 3A–C). Continuous stimulation (CS) was not effective in stimulating CO_2_ production, but it effectively increased the accumulation of the ASMs, which resulted in an elevated rate of total FOx (Fig. 3A–C). It is important to say that continuous stimulation tended to lower incorporation of ^14^C‐palmitate into muscle triacylglycerols and phospholipids, while the regulation was not observed in myotubes subjected to intermittent stimulation (Fig. 3D–F).

### Effects of electrical pulse stimulation on mitochondrial OxPHOS proteins and muscle fiber‐type markers of differentiated human muscle cells

**Fig. 4:**
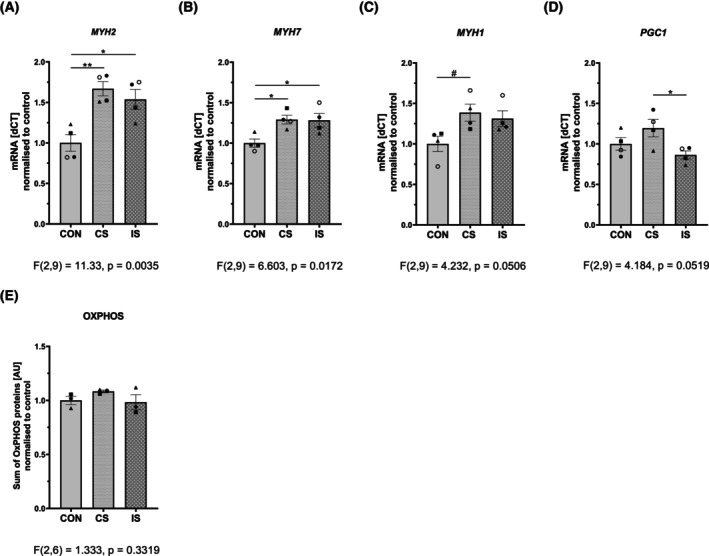
Effects of electrical pulse stimulation (EPS) on markers of fiber‐type, mitochondrial biogenesis, and content in primary human myotubes. Differentiated human muscle cells were exposed to continuous and intermittent EPS for 24 h. Gene expressions of (A) MYH2, marker of type IIa/fast‐twitch oxidative fibers; (B) MYH7, marker of type I/slow‐twitch oxidative muscle fibers; (C) MYH1, marker of type IIx/fast‐twitch glycolytic fibers; and (D) PGC1α, marker of mitochondrial biogenesis were determined. (E) Total protein content of the five mitochondrial OxPHOS complexes, a surrogate marker of mitochondrial content. Data are normalized to controls and presented as means ± SEM of *n* = 4 (3 independent experiments, one donor in duplicate). Individual donors were depicted by different symbols ▲, ●, ■. Empty symbol ○ indicates duplicate measurement in cells from the donor ●. The difference between control and EPS‐treated myotubes was assessed by one‐way ANOVA with Tukey's multiple comparison test. Level of significance: **P < 0.01, *P < 0.05 and *P* < 0.1 indicates trend. CON, control; CS, continuous EPS; IS, intermittent EPS; MYH, myosin heavy chain; OxPHOS, oxidative phosphorylation; PGC1α, peroxisome proliferator‐activated receptor gamma coactivator 1‐alpha.

Electrical pulse stimulation to primary muscle cells from metabolically healthy individuals elicited a shift towards a more oxidative muscle cell phenotype. This was evidenced by increased expression of Myosine Heavy Chain 2 (*MYH2*, fast‐twitch oxidative type IIa fibers marker) and Myosine Heavy Chain 7 (*MYH7*, slow‐twitch oxidative type I fibers marker) by both IS and CS, while the regulation of *MYH1* (marker of type IIx fast‐twitch glycolytic muscle fibers) was less affected (Fig. 4A–C). It is important to note that neither IS nor CS elicited any regulation of the transcription coactivator *PGC1α* mRNA (master regulator of mitochondrial biogenesis) (Fig. 4D). Similarly, the protein levels of specific OxPHOS complexes embedded in the inner mitochondrial membrane (Fig. S1), as well as the combined protein signal of all five OxPHOS complexes (surrogate marker of mitochondrial content) were not modulated by CS or IS (Fig. 4E).

### Effects of EPS on expression and secretion of muscle contraction‐induced myokines

**Fig. 5:**
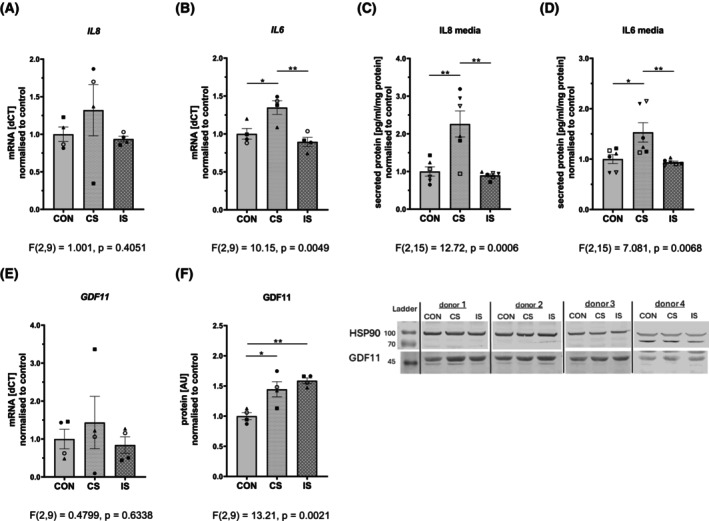
Effects of electrical pulse stimulation (EPS) on gene expression, protein content, and secretion of contraction‐induced myokines. Differentiated human muscle cells were exposed to EPS for 24 h. Gene expression of (A) IL8, (B) IL6, and (E) GDF11 is shown. Figures (C) and (D) depict protein content of IL8 and IL6 in EPS‐conditioned media, while (F) shows GDF11 protein content in EPS‐treated myotubes. The complete blots of GDF11 and HSP90 are presented in Fig. S2. Data are normalized to control and presented as means ± SEM, representing *n* = 4–6 (3 to 4 independent experiments with1–2 donors with duplicate measurement). Individual donors were depicted by different symbols ▲, ○, ●, ■, □, ▼. The difference between control and EPS‐treated myotubes was assessed by one‐way ANOVA with Tukey's multiple comparison test. Level of significance: **P < 0.01, *P < 0.05. CON, control; CS, continuous EPS; GDF11, growth differentiation factor 11; IL, interleukin; IS, intermittent EPS.

Gene expression of *IL6*, well‐known contraction‐induced myokine, was increased by continuous but not by intermittent EPS, while expression of *IL8* was not regulated, likely due to high interindividual variability. Changes in gene expression were well reflected in the release of the two myokines into the conditioned media (Fig. 5A–D). Next, we measured the EPS‐induced regulation of GDF11 gene and protein levels. The high interindividual variability blunted the effect of CS on GDF11 gene expression (Fig. 5E); however, we clearly observed an increase in GDF11 protein content in response to both stimulation protocols when compared to control, unstimulated myotubes (Fig. 5F; Fig. S2). We were unable to detect GDF11 in the conditioned media.

### Effects of EPS on extracellular vesicles from the conditioned media of human myotubes

**Fig. 6:**
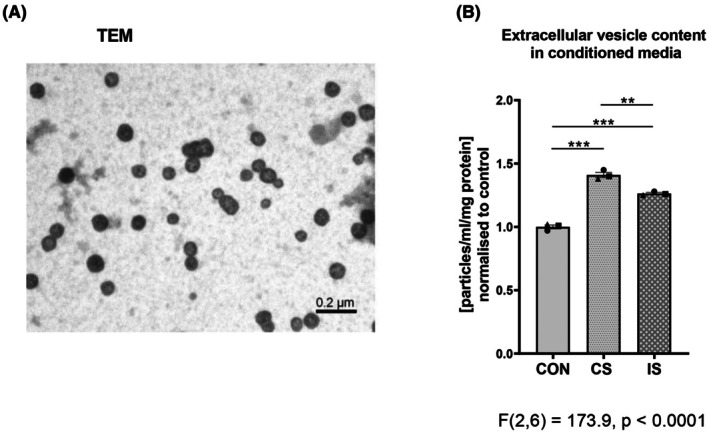
Effects of electrical pulse stimulation (EPS) on the content of extracellular vesicles (EVs) released from primary human myotubes. Human primary myotubes were exposed to EPS for 24 h, conditioned media were collected, and EVs were isolated by ExoQuick. (A) TEM image of EVs at 60000× magnification, (B) effect of continuous (CS) and intermittent (IS) EPS on the concentration of EVs isolated from EPS‐conditioned media as measured by nanoparticle tracking analysis. Data are normalized to control and presented as means ± SEM, representing *n* = 3 independent experiments using 3 different donors, as depicted by symbols ▲, ●, ■. The difference between control and EPS‐treated myotubes was assessed by one‐way ANOVA with Tukey's multiple comparison test. Level of significance: ***P < 0.001, **P < 0.01. CON, control; CS, continuous EPS; IS, intermittent EPS; TEM, transmission electron microscopy.

The extracellular vesicles (EVs) were characterized by transmission electron microscopy (TEM) and nanoparticle tracking analysis (NTA), and the presence of exosomal markers (subpopulation of EVs involved in organ crosstalk) was identified by the proteomic analysis. TEM revealed the presence of ~100 nm spherical structures with a well‐defined membrane (Fig. 6A).

The proteomic analysis of the EPS‐conditioned EVs identified 4164 proteins, 3953 of which are contained in either VesiclePedia or ExoCarta. However, due to the pilot nature of the experiment, it was not possible to obtain conclusive data on the specific effects of the two EPS protocols on the EVs proteome (2 replicates for each EPS protocol). It was, however, used to show the enrichment of exosomal proteins, including tetraspanins CD63, CD81, and CD9, and EVs cargo proteins TSG101, Alix, as well as several subunits of the mitochondrial ATP synthase in all the EVs samples. It is also important to say that neither of the examined myokines (IL6, IL8, GDF11) was identified as a part of EVs cargo. Data from this pilot proteomic analysis have been deposited to the ProteomeXchange Consortium via the PRIDE [35] partner repository, with the dataset identifier PXD059967.

Next, we performed NTA demonstrating that during 24 h and without any external stimuli, differentiated human myotubes released a substantial amount of EVs (nanoparticles 30–150 nm in diameter) into the media (3.1 × 10^11^ EVs/mL/mg of cell proteins). Importantly, both EPS protocols increased EVs content in the conditioned media (CS: 41%; IS 26%) (Fig. 6B). After 24 h, the stimulation concentration reached 3.9–4.4 × 10^11^ EVs/mL/mg of cell proteins. This result suggests that EPS‐induced contractile activity of myotubes *in vitro* enhances EVs' release into the conditioned media.

## Discussion

To date, many reports provide evidence that different types of EPS protocols have the potential to induce specific molecular adaptations and modulate energy metabolism and secretory profiles in primary cultures of human myotubes obtained from patients with obesity, type 2 diabetes, or cancer [12, 13, 17, 18, 22, 36, 37, 38]. Important impulse for this work was a report by Schwappacher *et al*. who performed whole‐body electrostimulation training in patients with prostate cancer to show that this stimulation, as well as the identical EPS protocol (2× 20 min lasting bouts of 6‐s stimulation, 4‐s pause at 85 Hz pulse frequency, 400 ms pulse width, 15 V amplitude within 24 h period) used in primary human muscle cells, produced the molecular cocktail with the capacity to lower the proliferation of cancer cells [39]. In our research, we use the tools of integrative physiology to study the effects of exercise in patients with obesity, type 2 diabetes, myopathies, mild cognitive impairment, and cancer [40, 41, 42, 43], as well as in healthy individuals [44, 45], focusing on the exercise‐regulated molecules potentially involved in the systemic integration of the adaptive response to exercise. The ultimate goal of this study was to develop an EPS protocol that would better reflect the episodic nature of repeated exercise bouts *in vivo*, and therefore induce physiologically and metabolically relevant responses in cultured human myotubes, including their secretory function. The primary objective was to examine changes in energy metabolism and secretory function of muscle cells derived from healthy individuals, subjected to the newly designed EPS protocol, alternating longer periods of subthreshold electrical stimulation (4 h resting periods) with shorter periods of higher frequency stimulation (30‐min exercise periods). A commonly used continuous low‐frequency (1 Hz, 24 h) EPS protocol was used as a comparator. Intensive contraction step design in our intermittent stimulation protocol was inspired by the work by Nicolić *et al*. [7]. They examined the metabolic effects of 60‐min intermittent EPS stimulation where trains of bipolar pulses (2, 5, 10, 100 Hz, 200 ms) were given every 5th second and voltage was set to 30 V. Results showed that maximum deoxyglucose uptake was provided by stimulation at 2–5 Hz, and that optimal effects on lactate release and on stabilization of phosphocreatine/ATP exchange supporting muscle contractions occurred after 30 min of stimulation [7]. This type of stimulation was further tested because it seemed to mimic a response similar to a single bout of exercise *in vivo*.

First, we examined EPS‐induced changes in glucose and fatty acid metabolism. We showed that intermittent (IS) but not continuous (CS) stimulation increased glucose oxidation in myotubes. Incorporation of ^14^C‐glucose into glycogen as well as total glucose disposal increased irrespective of the EPS protocol used, and a similar effects were observed both with and without insulin. Previous reports using low frequency (0.1 and 1 Hz) chronic (24 h and 48 h) EPS reported an increase in both oxidative [7, 8, 12, 18] and nonoxidative [7, 8, 22] glucose metabolism or glucose uptake in primary myotubes from metabolically healthy donors. Our results clearly show superior efficiency of intermittent compared to continuous stimulation with respect to the oxidative capacity of muscle cells. The fact that only intermittent EPS increased glucose oxidation, and that neither of the two EPS protocols stimulated oxidative glucose metabolism in response to insulin, while preferring glycogen synthesis for glucose disposal, clearly indicates that energy requirements at the time of cell harvest, that is, 3 h after the last intensive contractile activity period (IS), and at the end of low‐intensity chronic stimulation (CS) were low. There is a large heterogeneity in the effects of continuous EPS on glucose metabolism in the literature. While Park *et al*. [22] reported that insulin treatment is necessary to reveal effects of EPS (1 Hz, 11.5 V, 2 ms, 24 h) on glucose metabolism, Feng *et al*. [18] found that continuous EPS (1 Hz, 30 V, 2 ms, 48 h) stimulated glucose oxidation but failed to enhance glycogen synthesis in myotubes from lean donors irrespective of the insulin treatment. Nicolic *et al*. [7] showed that 5 Hz stimulation quickly (within minutes) stimulated deoxyglucose uptake, but metabolic deceleration was not examined.

Next, we observed that intermittent stimulation of human primary myotubes enhanced both complete (CO_2_ production) and incomplete (intermediate metabolites of FOx) fatty acid oxidation, thus increasing the total fatty acid disposal rate. However, continuous stimulation was not effective in inducing CO_2_ production, but it effectively increased incomplete FOx, which resulted in an elevated fatty acid disposal. The effects of EPS on fatty acid oxidation are contradictory in the literature. While some studies reported increased uptake [12] and oxidation of oleic [7, 12, 18] and palmitic acid [27, 28] after low frequency (0.1 or 1 Hz, 10 V, 2 ms) 24 h or 48 h EPS, others found no change in oleate and palmitate oxidation (1 Hz, 11.5 V or 30 V, 2 ms; 24 h) [7, 8] in primary human muscle cells. It is plausible to think that a relatively small effect of EPS on FOx in myotubes is explained by the fact that human primary myotubes in culture rely predominantly on glucose metabolism, as they express more glycolytic fiber‐type characteristics [7, 46, 47]. The superior efficiency of IS compared to CS in increasing CO_2_ production from both glucose and fatty acids supports the hypothesis that intermittent stimulation, which alternates episodes of stimulation and regeneration, exerts a greater adaptive effect on the oxidative capacity of muscle cells as compared to exhaustive continuous stimulation.

Continuous but not intermittent stimulation lowered or tended to lower incorporation of ^14^C‐palmitate into phospholipids (PL) and triacylglycerols (TAG). This suggests that CS may shift fatty acids towards incomplete FOx (accumulation of intermediate metabolites), and away from TAG and PL synthesis. Our findings partially contrast with a previous study by Løvsletten *et al*. [28] who reported that 24 h EPS (0.1 Hz, 10 V, 2 ms) increased FA incorporation into PL without affecting DAG and TAG pools. Feng *et al*. [18] found no changes in lipid droplet accumulation in muscle cells in response to 48 h low‐frequency EPS. These differences could stem from the specific details in EPS protocols (0.1 Hz vs. 1.0 Hz, 24 h vs 48 h stimulation) as well as from the distinct responsiveness of myotubes derived from donors with specific clinical phenotypes (lean healthy donors vs individuals with obesity and/or type 2 diabetes).

Adaptations that promote muscle energy metabolism during exercise often involve mitochondria and specific changes in fiber type. We observed that both CS and IS increased expression of *MYH2* and *MYH7*, markers of fast‐ and slow‐twitch oxidative muscle fibers. However, expression of markers of fast‐twitch glycolytic fibers (*MYH1*), transcriptional coactivator regulating mitochondrial biogenesis (*PGC1a*) and OxPHOS proteins indicating mitochondrial content were not regulated by 24 h EPS in this study. This might indicate that both EPS protocols initiated a shift in cultured human muscle cells toward a more oxidative phenotype, but the structural and functional adaptations were not completed after 24 h stimulation. Nikolić *et al*. [7] found that continuous EPS (1 Hz, 30 V, 2 ms, 24 and 48 h) induced a 45% increase in protein expression of a marker of type I (slow oxidative) fibers, accompanied by elevated mitochondrial content, but without significant upregulation of PGC1α, in human primary myotubes. In contrast, Lambernd *et al*. [8] observed an increase in MYH1 mRNA, a marker of fast glycolytic fibers, without changes in *MYH2* expression. Interestingly, this was accompanied by elevated levels of mitochondrial OxPHOS complexes II, IV, and V by a comparable stimulation pattern (1 Hz, 11.5 V, 2 ms, for 24 h). In other studies involving continuous EPS (1 Hz, 30 V, 2 ms for 48 h), no changes were observed in gene and protein expression of MYH7, a marker of type 1 slow‐twitch oxidative fibers, while there was an increase in mitochondrial abundance in myotubes from lean, metabolically healthy individuals [18, 48]. In contrast, studies using lower‐frequency stimulation (0.1 Hz, 10 V, 2 ms, for 24 h) found minimal or no changes in OxPHOS proteins, suggesting that the intensity and frequency of EPS could be critical for eliciting mitochondrial adaptations [12, 28].

Skeletal muscle responds to exercise by releasing the entire spectrum of bioactive molecules known as myokines [49]. Myokines play a key role in the integration of the exercise‐induced systemic adaptations. Here, we show specific regulation of genes encoding selected myokines (IL6, IL8 and GDF11) in muscle cells exposed to EPS and their release into the conditioned media. Expression of *IL6* was increased by continuous but not by intermittent stimulation, while expression of genes encoding *IL8* and *GDF11* was not regulated in muscle cells by EPS treatment. CS but not IS led to robust accumulation of IL6 and IL8 in the conditioned media, while GDF11 protein levels in myotubes increased in response to both EPS protocols. Similar low‐frequency EPS (1 Hz, 30 V, 2 ms) lasting 48 h induced IL6 but not IL8 gene expression in myotubes derived from lean donors and donors with obesity [18]. Release of IL6 [8, 13] and IL8 [12, 13] was confirmed in several other studies using low‐frequency continuous EPS (1 Hz, 11.5 V, 2 ms). Moreover, IL6 gene expression was increased after both short (3 h, 0.5 Hz, 10 V, 24 ms pulses, acute intense exercise model) and prolonged (24 h, 0.1 Hz, 10 V, 2 ms pulses, chronic moderate exercise model) EPS [27]. We confirmed the effectiveness of CS to induce expression and secretion of well‐known contraction‐stimulated myokines. Harvesting the cells 3 h after the last 5 Hz stimulation period, during the low‐frequency (0.2 Hz) stage, could explain a lack of effect in the intermittent EPS on IL6 and IL8 in our model.

GDF11 is a protein from the transforming growth factor β (TGF‐β) family, shown to restore the regenerative potential of skeletal muscle, heart, and brain [20, 50, 51]. Previously, we described the modulating effect of acute intensive aerobic exercise (90 min run) on GDF11 levels in cerebrospinal fluid but not in the systemic circulation of healthy young trained individuals [21]. Demontis *et al*. [19] showed that overexpression of myoglianin, a myokine orthologous to human GDF11 and myostatin, extends lifespan in drosophila. Long‐term exercise training is associated with higher circulating levels of GDF11 [52, 53, 54] and increased expression in the skeletal muscle of mice [55, 56]. Here we detected an increase in GDF11 protein in myotubes subjected to both EPS protocols, supporting the regulatory role of muscle contraction in GDF11 expression. However, we did not detect GDF11 in the cell culture media, so we were not able to confirm that GDF11 is an exercise‐regulated secretory product of muscle cells (myokine).

Exercise regulates the release of extracellular vesicles (EVs) and their cargo of bioactive molecules, with a capacity to influence the integrative adaptive response via cellular and organ crosstalk [57, 58, 59]. We isolated EVs from cell culture media of human primary myotubes, characterized them in accordance with MISEV 2023 [60] and subjected them to proteomic analysis to check the feasibility of this approach for studying exosomes, the subpopulation of EVs involved in interorgan crosstalk, and to examine the presence of selected myokines (IL6, IL8, GDF11). Morphological features, such as the presence of 70–130 nm spherical vesicles surrounded by a lipid bilayer and a positivity of exosomal markers CD63, CD81, TSG101, and Alix, indicate the presence of exosomes in our EV samples. NTA demonstrated the release of a substantial amount of EVs (nanoparticles 30–150 nm in diameter) into the cell culture media by myotubes at baseline unstimulated state (3.1 × 10^11^ EVs/mL/mg of cell proteins). Importantly, both EPS protocols induced a 41% (CS) and 26% (IS) increase in the nanoparticle content in the conditioned media, indicating that EPS directly enhances EV release from contracting muscle cells *in vitro*. Proteomic analysis of EPS‐induced EVs identified 4164 proteins, 3953 of which are contained in either VesiclePedia or ExoCarta (PRIDE repository, project accession# PXD059967). However, neither of the selected myokines (IL6, IL8, GDF11) were identified in EV cargo. Our findings are consistent with previous studies using murine C2C12 myotubes. Murata *et al*. [15] demonstrated that tetanic EPS (30 Hz, 1 s, 10 V, 2 ms pulse, altering with 39 s rest, for 24 h) increased both the release of EVs and the expression of EV marker protein (Alix). In the same model, continuous low‐frequency stimulation (1 Hz for 24 h) did not enhance EV release [15]. Similarly, during chronic contractile activity (CCA; 1 Hz, 14 V, 2 ms 3 h/day for 4 days), the concentration of EVs and their markers (CD81, CD63, TSG101) progressively increased over time [61]. To date, only one study has examined EPS‐induced EV release in primary human myotubes. In this study, Aas *et al*. [14] reported that EPS (1 Hz, 30 V, 2 ms for 24 h) did not significantly alter EV concentration. However, EPS significantly modified the molecular composition of EVs, with changes in 15 exosomal miRNAs and alterations in the protein content of both exosomes (73 proteins) and microvesicles (97 proteins) derived from myotubes of females with grade III obesity and type 2 diabetes [14]. Our results expand on these findings and provide further evidence that EPS can stimulate EV secretion in primary human myotubes. This highlights EPS as a suitable *in vitro* model for studying the exercise‐related EV secretome.

## Conclusions

In this study, we examined the effects of intermittent and continuous electrical pulse stimulation protocols on energy metabolism and secretory function of primary human differentiated muscle cell cultures. We showed that intermittent high‐frequency EPS elicited a stronger adaptive response in the capacity of myotubes to oxidize glucose and fatty acids, an adaptation allowing effective energy production, as compared to low‐frequency continuous stimulation. However, continuous but not intermittent stimulation elicited a consistent increase in specific contraction‐induced myokines in the conditioned media, indicating the importance of timing for the conditioned media harvest. Evidence on the contraction‐induced regulation of the putative myokine, GDF11, and on the release of extracellular vesicles into the media in response to both continuous and intermittent stimulation highlights human primary myotubes as a model suitable for characterizing exercise‐regulated muscle‐derived proteins and EVs, and their role in muscle and integrative exercise physiology.

Our findings confirm prior observations on EPS‐induced metabolic remodeling and myokine regulation, while offering novel insights into the differential responses elicited by intermittent versus continuous stimulation. The application of the novel intermittent EPS protocol in primary human myotubes advances current *in vitro* exercise models by more closely mimicking the episodic nature of *in vivo* muscle contraction and capturing relevant metabolic and EVs secretory adaptations.

### Study limitations

The phenotypes of myotubes were explored at specifically selected time points, precluding us from observing the dynamics of expression regulation and the cell secretory patterns across the wider time frame. Relatively large variability in the EPS‐induced response (GDF11, IL8 mRNA) could be, to an extent, attributable to the interindividual differences in the clinical phenotypes of the cells' donors, including family history of obesity and type 2 diabetes. The study was conducted using a relatively small number of biological replicates, which could reduce the capacity to detect all the adaptive responses to electrical pulse stimulation.

## Conflict of interest

The authors declare no conflict of interest.

## Authors contributions

Optimization of EPS protocols, cell culture studies, data analyses, and drafting the manuscript (KG, TK); data interpretation, conceptualization of the manuscript (TK, BU); EVs isolation, characterization, and content analysis of the EV data (DB, ST); fatty acid and glucose oxidation assays (TK, KG, NP); cell culture studies (JB); transmission electron microscopy (MN); thin layer chromatography (BM); proteomic analysis (VP); statistical analysis (MS); conception and design of the study, data interpretation, and revising the manuscript critically for important intellectual content (BU, JU); all authors provided approval of the final version of the manuscript.

## Supporting information


**Fig. S1.** Immunoblots and graphs of individual proteins specific for the mitochondrial OxPHOS complexes.
**Fig. S2.** Immunoblots of growth differentiation factor 11 and heat shock protein 90 housekeeper in muscle cell lysates.
**Table S1.** Information on number of technical replicates.
**Table S2.** List of primer pairs.

**Video S1.** Continuous electrical pulse stimulation.

**Video S2.** Intermittent electrical pulse stimulation.

## Data Availability

The data that support the findings of this study are available from the corresponding author upon reasonable request. Data of the pilot proteomic analysis have been deposited to the ProteomeXchange Consortium via the PRIDE [35] partner repository, with the dataset identifier PXD059967.
