# Supplementary material for: Electrical pulse stimulation reflecting the episodic nature of real‐life exercise modulates metabolic and secretory profile of primary human myotubes
Source: FEBS Open Bio. 2025 Sep 9;16(1):127–44. doi: 10.1002/2211-5463.70114 (PMC12767763; doi:10.1002/2211-5463.70114)
Supplement: Supplementary file 1 — Fig. S1. Immunoblots and graphs of individual proteins specific for the mitochondrial OxPHOS complexes. Fig. S2. Immunoblots of growth differentiation factor 11 and heat shock protein 90 housekeeper in muscle cell lysates. Table S1. Information on number of technical replicates. Table S2. List of primer pairs. [file FEB4-16-127-s002.docx]

**Supplementary data**

**Supplementary table 1.** Information on number of technical replicates.

| Batch No. | Examination |  | Cells  (Donor 1)  No of wells | Cells  (Donor 2)  No of wells | Cells  (Donor 3)  No of wells | Cells  (Donor 4)  No of wells |
| --- | --- | --- | --- | --- | --- | --- |
| 1. | Glucose metabolism (glucose oxidation, glycogen synthesis) | 12 well plate | 3, triplicate | 3, triplicate | 3, triplicate | EPS failure |
| 1. | Protein harvest  (Western blot) | 6 well plate | 2, duplicate | 1, monoplicate | 1, monoplicate | EPS failure |
| 1. | mRNA isolation  (qPCR) | 6 well plate | 2, duplicate | 1, monoplicate | 1, monoplicate | EPS failure |
| 2. | Lipid metabolism  (palmitate oxidation, lipid synthesis) | 12 well plate | 3, triplicate | 3, triplicate | 3, triplicate | 3, triplicate |
| 2. | Media harvest  (cell secretome) | 6 well plate | 1, monoplicate | 2, duplicate | 1, monoplicate | 2, duplicate |

**Supplementary table 2.** List of primer pairs. β2M - β2-microglobulin, GAPDH - glyceraldehyde 3-phosphate dehydrogenase, GDF11 - growth differentiation factor 11, IL6 - interleukin 6, IL8 - interleukin 8, MYH1 - myosin heavy chain 1, MYH2 - myosin heavy chain 2, MYH7 - myosin heavy chain 7, PGC1α - peroxisome proliferator-activated receptor gamma coactivator 1-alpha.

| **Gene** | **Primer sequences** | |
| --- | --- | --- |
|  | **Forward** | **Reverse** |
| ***PGC1α*** | TTACAAGCCAAACCAACAACTTTATC | CACACTTAAGGTGCGTTCAATAGTC |
| ***MYH1*** | TAAGACCGAGGCAAAAGGA | TGCATCAGCCAAGCTGTC |
| ***MYH2*** | TGTCTCACTCCCAGGCTACA | CCAAAAACAGCCAATTCTGAG |
| ***MYH7*** | CTTCGTGCCTGATGACAA | CATGTCTTGCCATA |
| ***IL6*** | AAAGAGGCACTGGCAGAAAA | TTTCACCAGGCAAGTCTCCT |
| ***IL8*** | GGCAGCCTTCCTGATTTC | TTGGAGTATGTCTTTATGCACTGACA |
| ***GDF11*** | GATCCTGGACCTACACGACTTC | GGCCTTCAGTACCTTTGTGAAC |
| ***β2M*** | CGCTCCGTGGCCTTAGC | AATCTTTGGAGTACGCTG |
| ***GAPDH*** | ACCAGGTGGTCTCCTCTGAC | TGCTGTAGCCAAATTCGTTG |


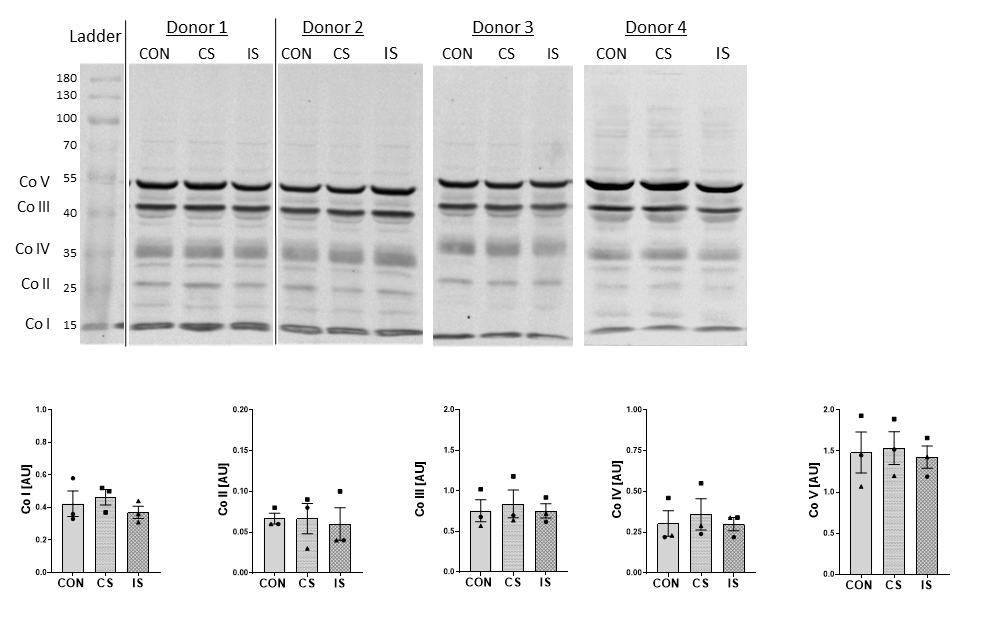


**Supplementary figure 1** Immunoblots and graphs of individual proteins specific for the mitochondrial OxPHOS complexes. CoI, complex I (subunit NDUFB8); CoII, complex II (subunit SDHB); CoIII, complex III (subunit UQCRC2); CoIV, complex IV (subunit MTCO1); CoV complex V (subunit ATP5A). Samples from 4 biological replicates were analysed in 2 separate membranes. The bands were cut out of the membranes that was not related to this article - the place of cutting marked with a black line. Data are normalised to the housekeeper protein HSP90 and shown as means ± SEM, representing n = 3 independent experiments, as depicted by different symbols ▲, ●, ■. The difference between control and EPS-treated myotubes was assessed by paired t-test. Level of significance: * p < 0.05. Co – complex, CON – control, CS - continuous EPS, EPS - electrical pulse stimulation, IS - intermittent EPS.

**
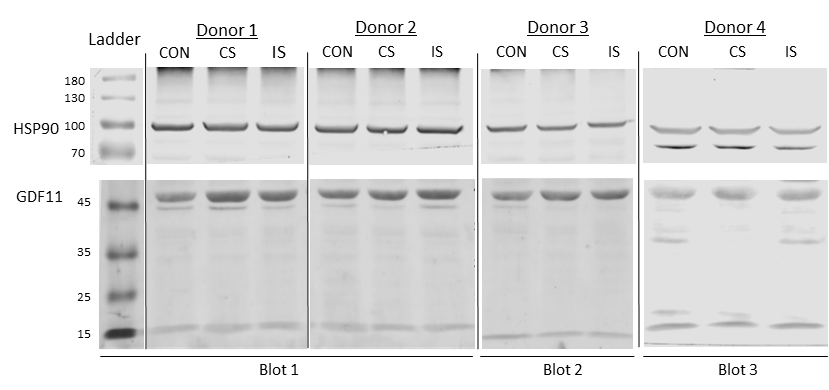
**

**Supplementary figure 2**. Immunoblots of growth differentiation factor 11 (GDF11) and heat shock protein 90 (HSP90) housekeeper in muscle cell lysates. Samples from 4 biological replicates were analysed in 2 separate membranes. The bands were cut out of the membranes that were not related to this article - the place marked with a black line. CON – control, CS - continuous EPS, EPS - electrical pulse stimulation, IS - intermittent EPS.
